# Supplementary material for: Synthesis, Optical Characterization in Solution and Solid-State, and DFT Calculations of 3-Acetyl and 3-(1′-(2′-Phenylhydrazono)ethyl)-coumarin-(7)-substituted Derivatives
Source: Molecules. 2022 Jun 8;27(12):3677. doi: 10.3390/molecules27123677 (PMC9227197; doi:10.3390/molecules27123677)
Supplement: Supplementary file 1 [file molecules-27-03677-s001.zip › molecules-1704102-supplementary.pdf]

Supplementary Information

Synthesis, optical characterization in solution and solid-state, and DFT calculations of 3-Acetil-coumarin-(7)-substituted derivatives.

Cesar A. Villa-Martínez[1], Nancy E. Magaña-Vergara[2*], Mario Rodríguez[3], Juan P. Mojica-Sánchez[4], Ángel A. Ramos-Organillo[1], Joaquín Barroso-Flores[5,6] I.I. Padilla-Martínez[7] and Francisco J. Martínez-Martínez[1*].

[1] Facultad de Ciencias Químicas, Universidad de Colima, Km 9 Carretera Coquimatlán-Colima, Coquimatlán Colima 28400, México. Email.: cvmartinez@ucol.mx

[2] CONACyT, Facultad de Ciencias Químicas, Universidad de Colima, Km 9 Carretera Coquimatlán-Colima, Coquimatlán Colima 28400, México

[3] Centro de Investigaciones en Óptica A. P. 1-948, 37000, León Gto. México.

[4] Tecnológico Nacional de México, Instituto Tecnológico José Mario Molina Pasquel y Henríquez Campus Tamazula de Gordiano, Carretera Tamazula-Santa Rosa No. 329, 49650 Tamazula de Gordiano, Jalisco, México

[5] Centro Conjunto de Investigación en Química Sustentable UAEM-UNAM, Unidad San Cayetano, Carretera Toluca-Atlacomulco Km.14.5, 50200, Toluca de Lerdo, México.

[6] Instituto de Química, Universidad Nacional Autónoma de México, Circuito Exterior, Ciudad Universitaria, Ciudad de México 04510, México.

[7] Laboratorio de Química Supramolecular y Nanociencias, Instituto Politécnico Nacional-UPIBI, Av. Acueducto s/n Barrio la Laguna Ticomán, Ciudad de México, C.P. 07340, México

Authors to whom correspondence should be addressed; e-mail: fjmartin@eucol.mx; Tel.: +52-312-3161163 and nancymv@ucol.mx.

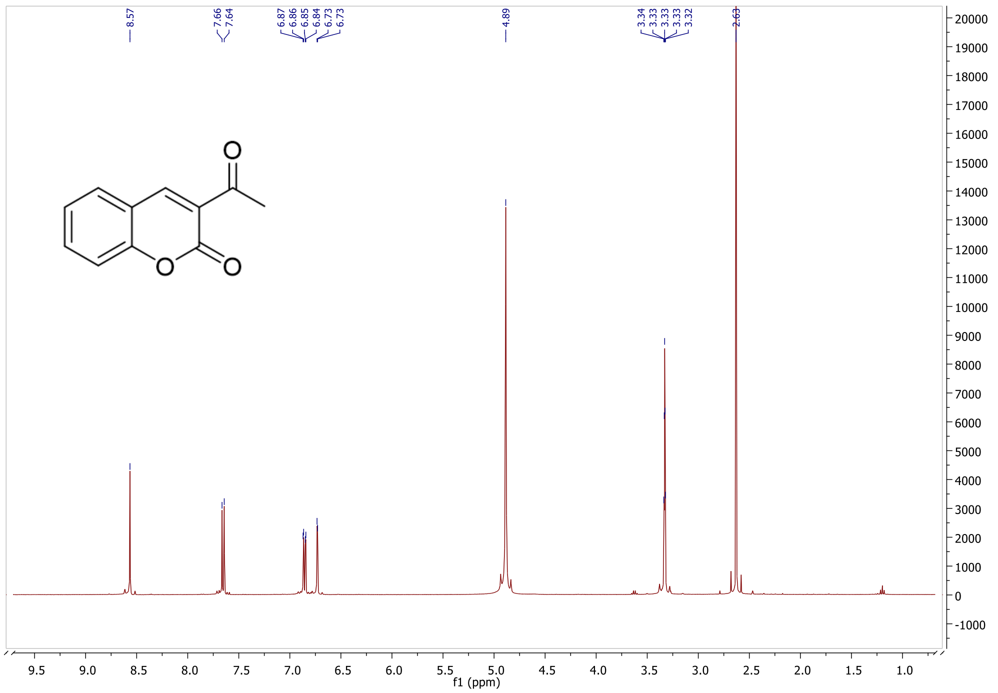

Figure S1. ^{1}H-NMR of compound 1a

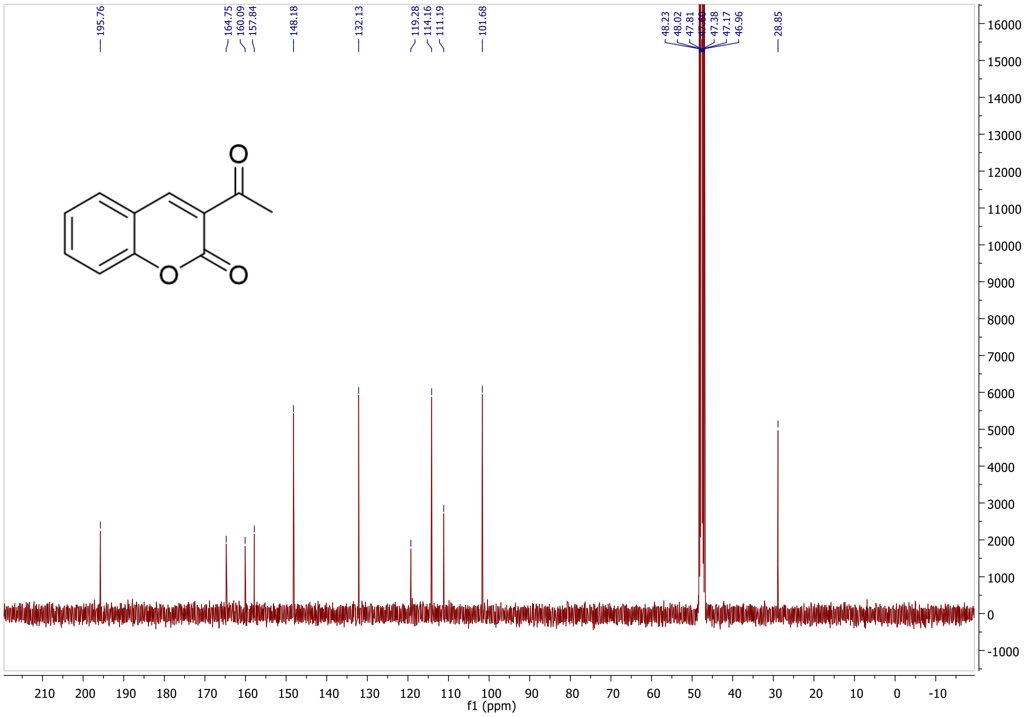

Figure S2. ^{13}C-NMR of compound 1a

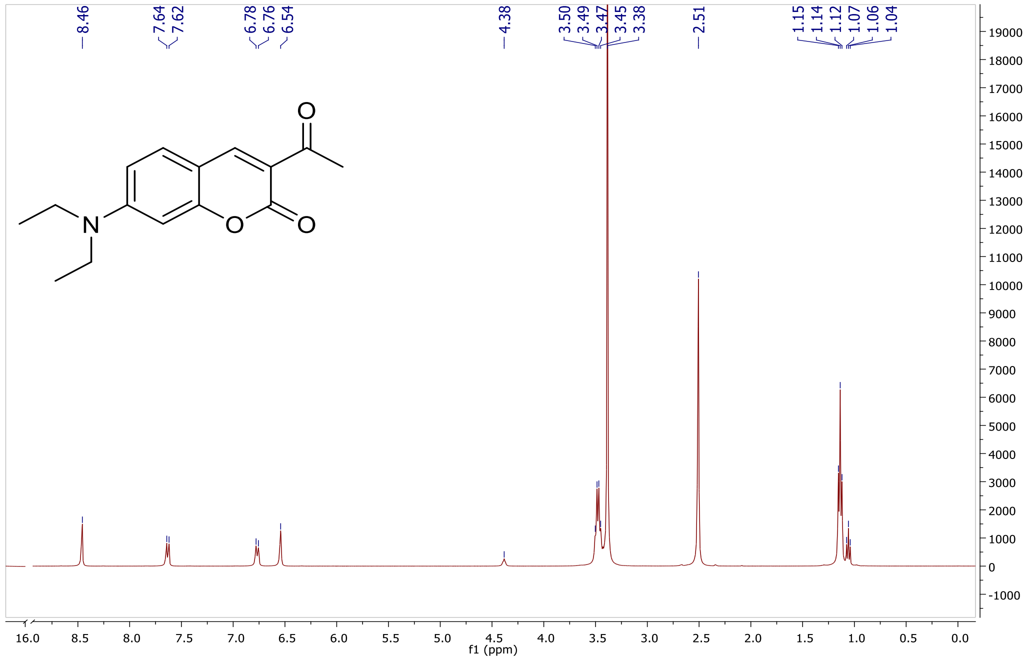

Figure S3. ^{1}**H-NMR of compound 1b**

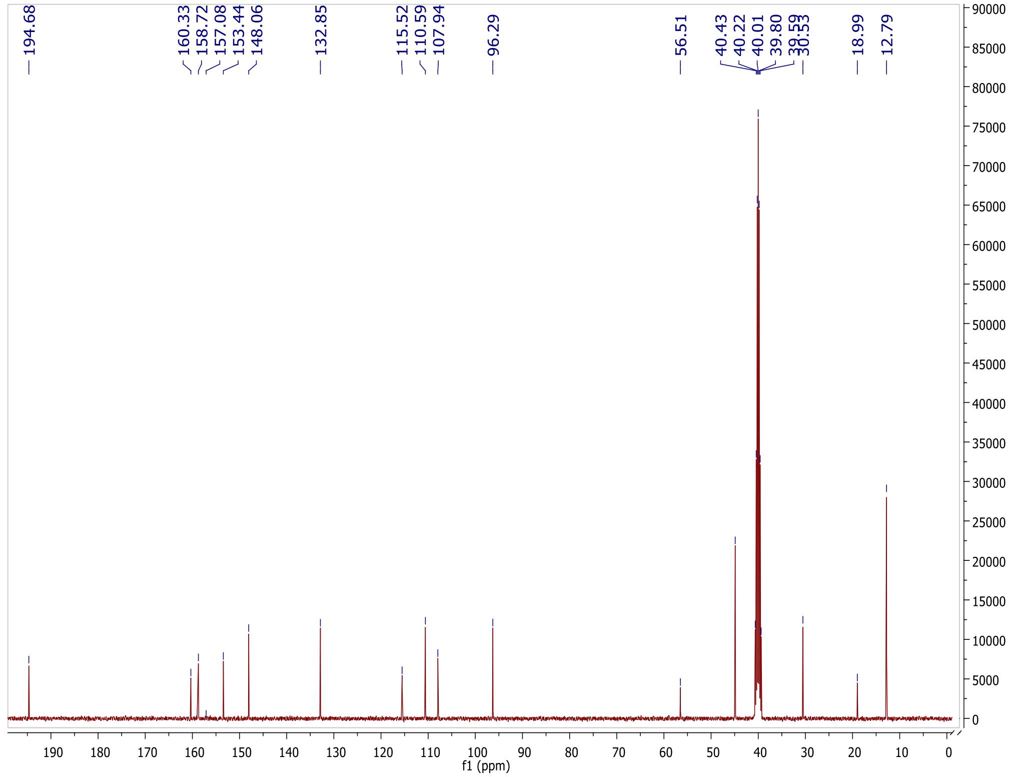

Figure S4. ^{13}**C-NMR of compound 1b**

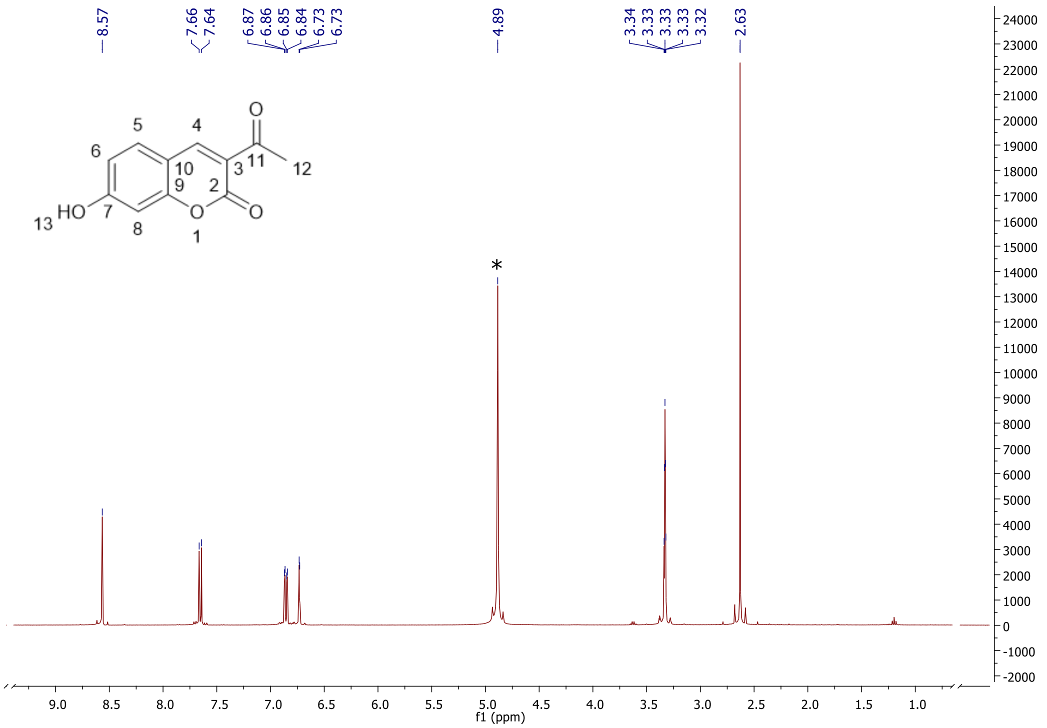

Figure S5. **^{1}H-NMR of compound 1c (* H_2O)**

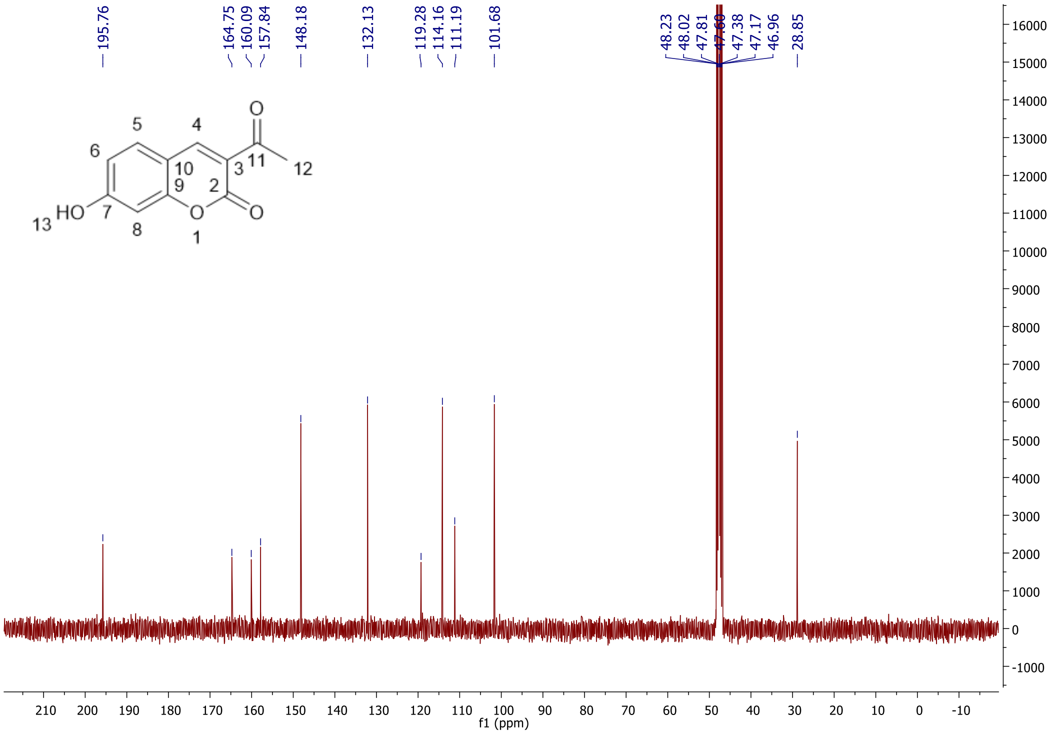

Figure S6. **^{13}C-NMR of compound 1c**

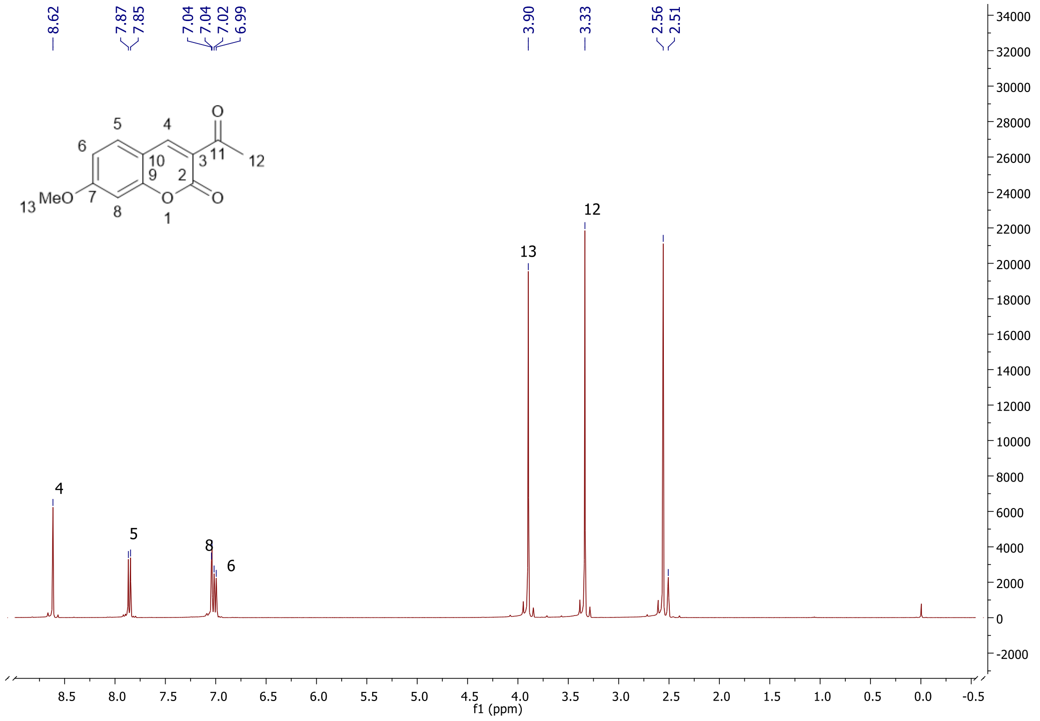

Figure S7. ^{1}H-NMR of compound 1d

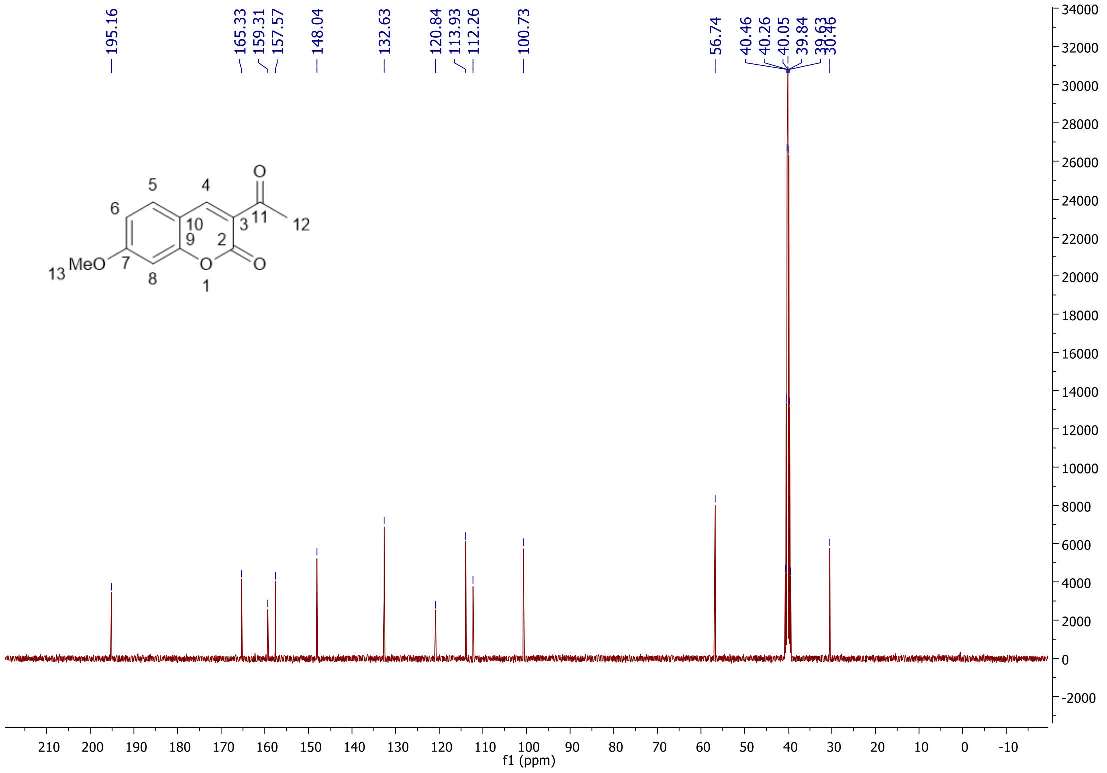

Figure S8. ^{13}C-NMR of compound 1d

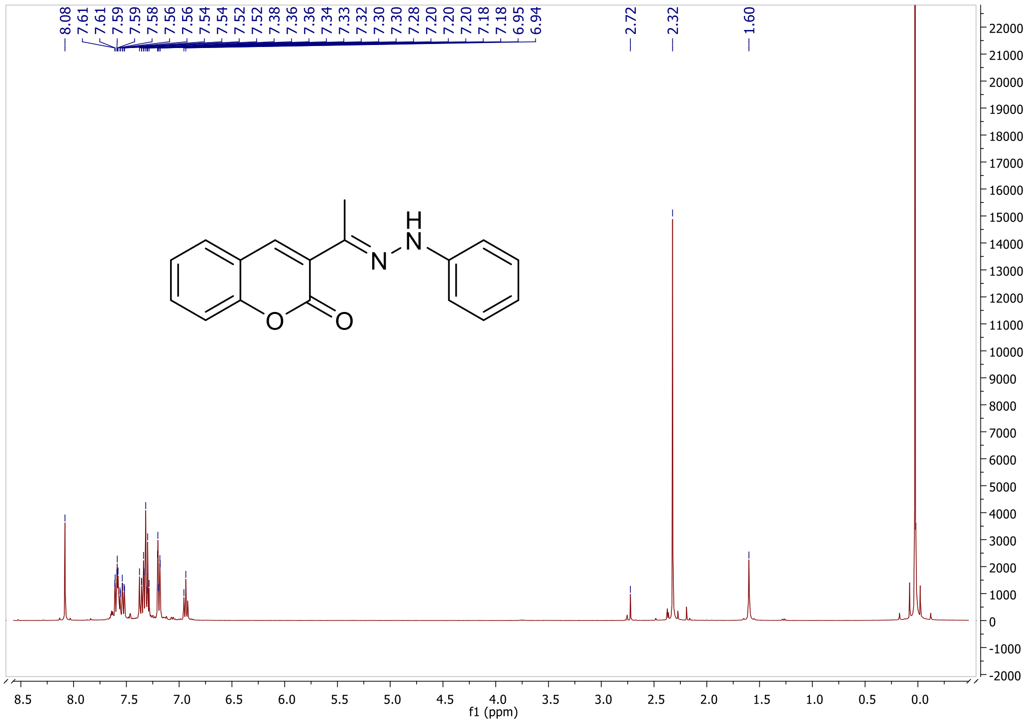

Figure S9. ^{1}H-NMR of compound 2a

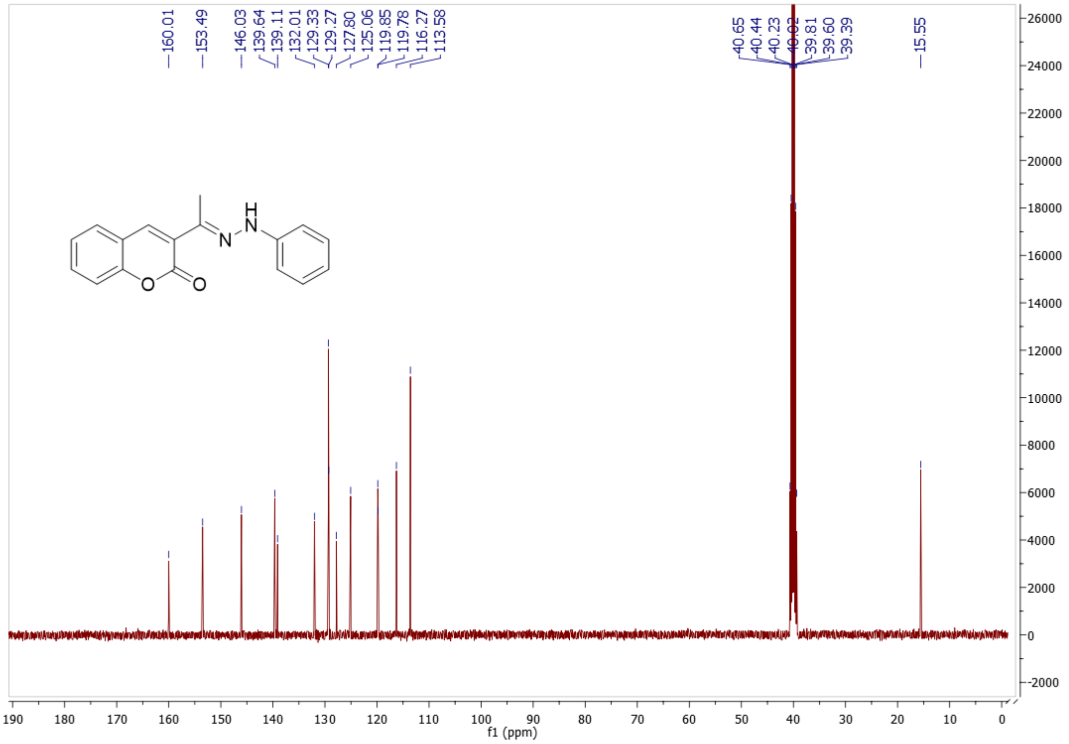

Figure S10. ^{13}C-NMR of compound 2a

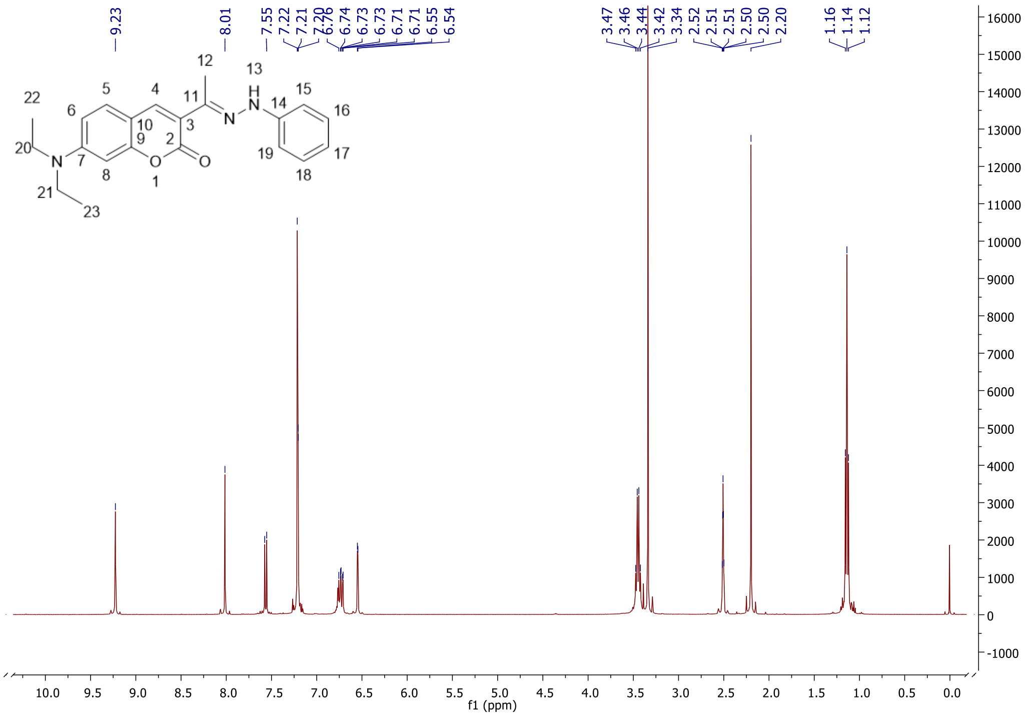

Figure S11. ^{1}H-NMR of compound 2b

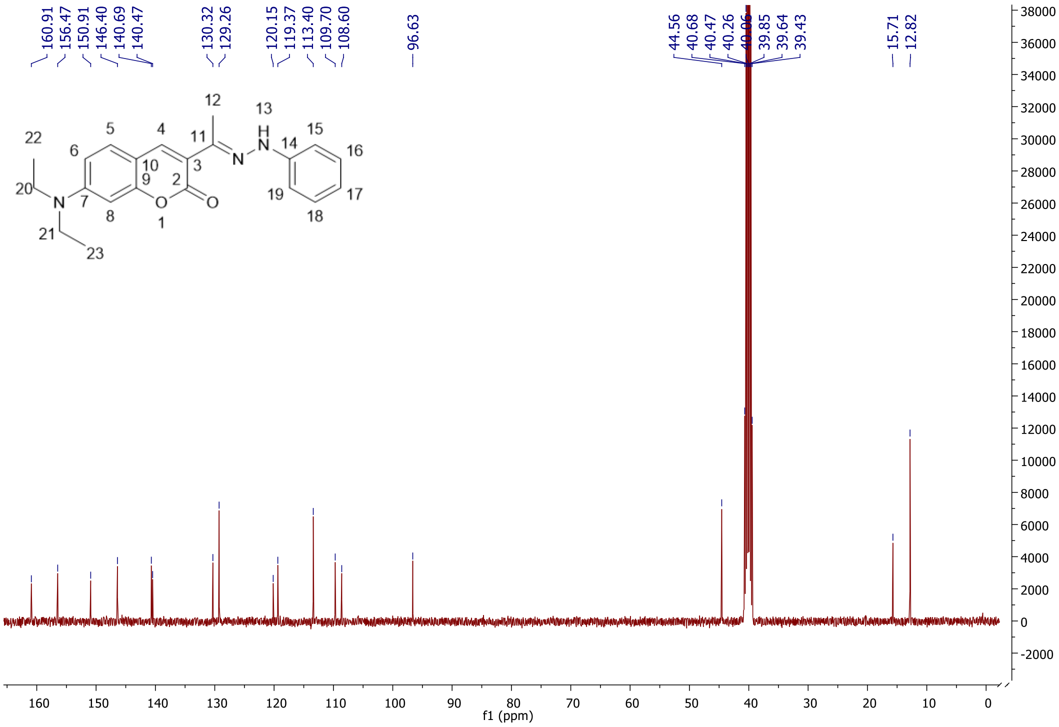

Figure S12. ^{13}C-NMR of compound 2b

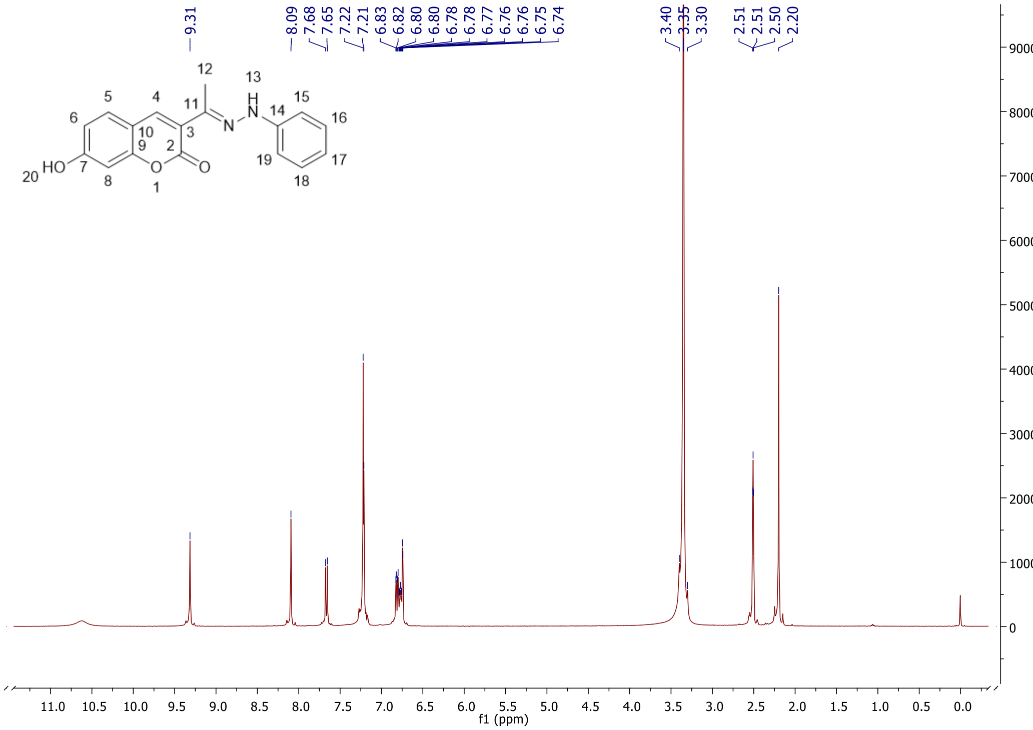

Figure S13. ^{1}H-NMR of compound 2c

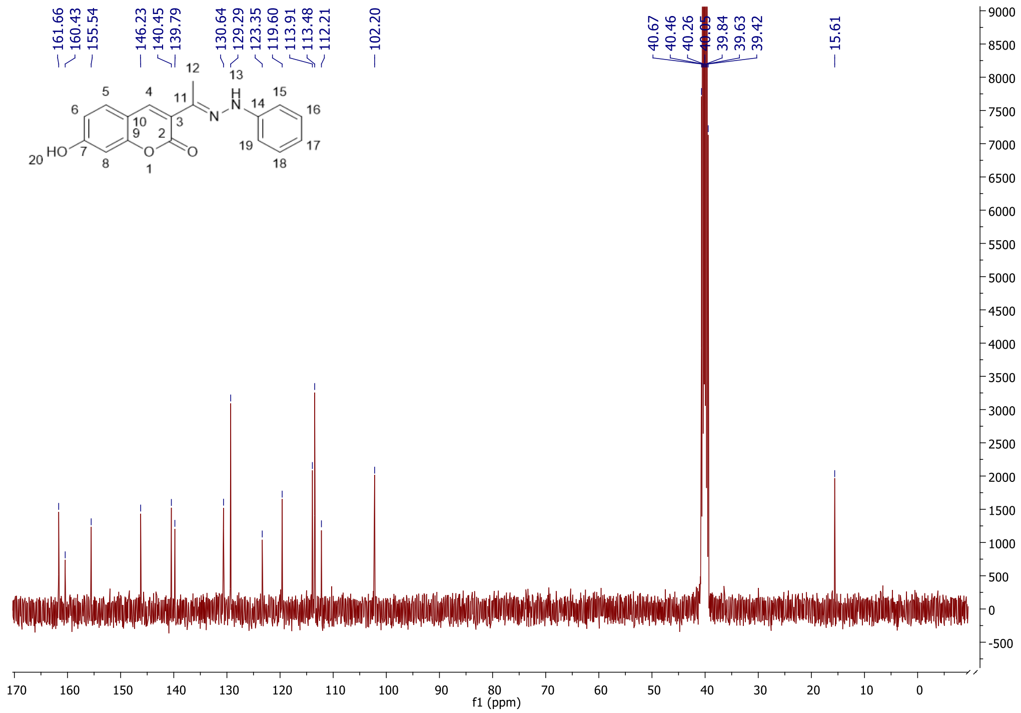

Figure S14. ^{13}C-NMR of compound 2c

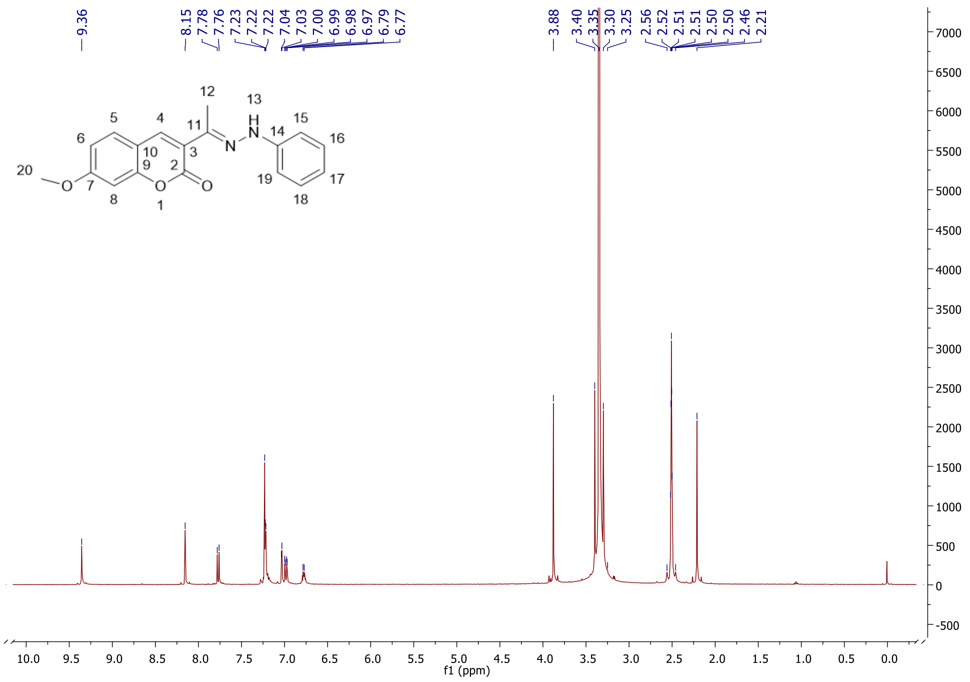

Figure S15. ^{1}H-NMR of compound 2d

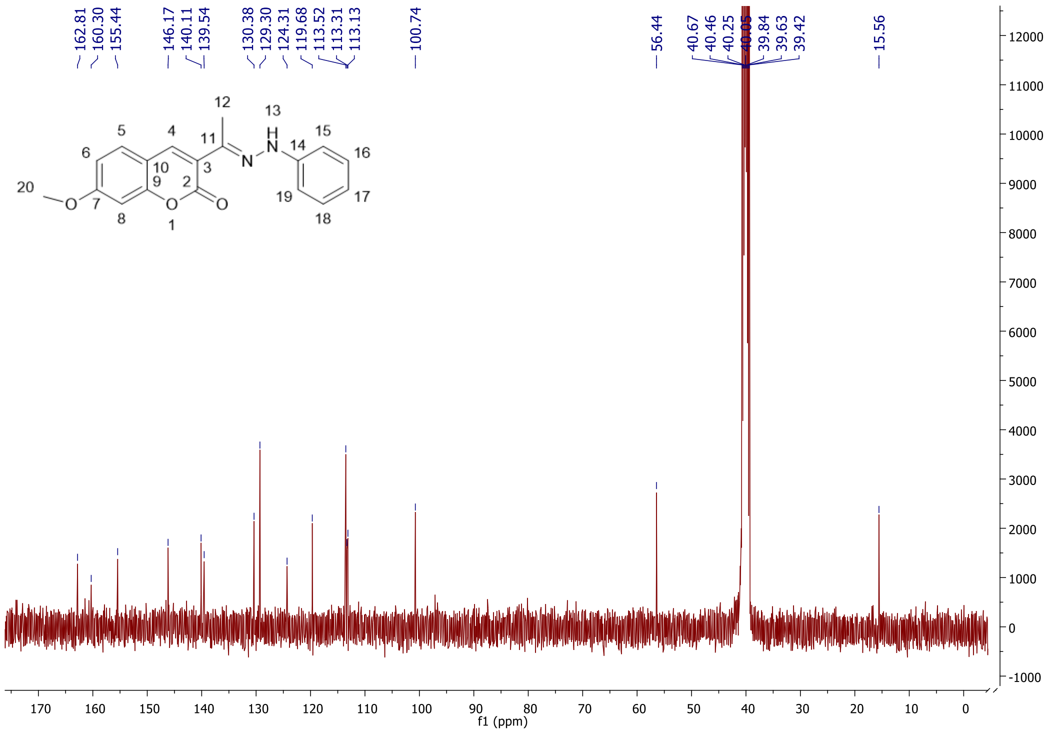

Figure S16. ^{13}C-NMR of compound 2d

Experimental (exp) and reference (ref) X-ray powder diffraction of compounds 1a, 1b and 1d

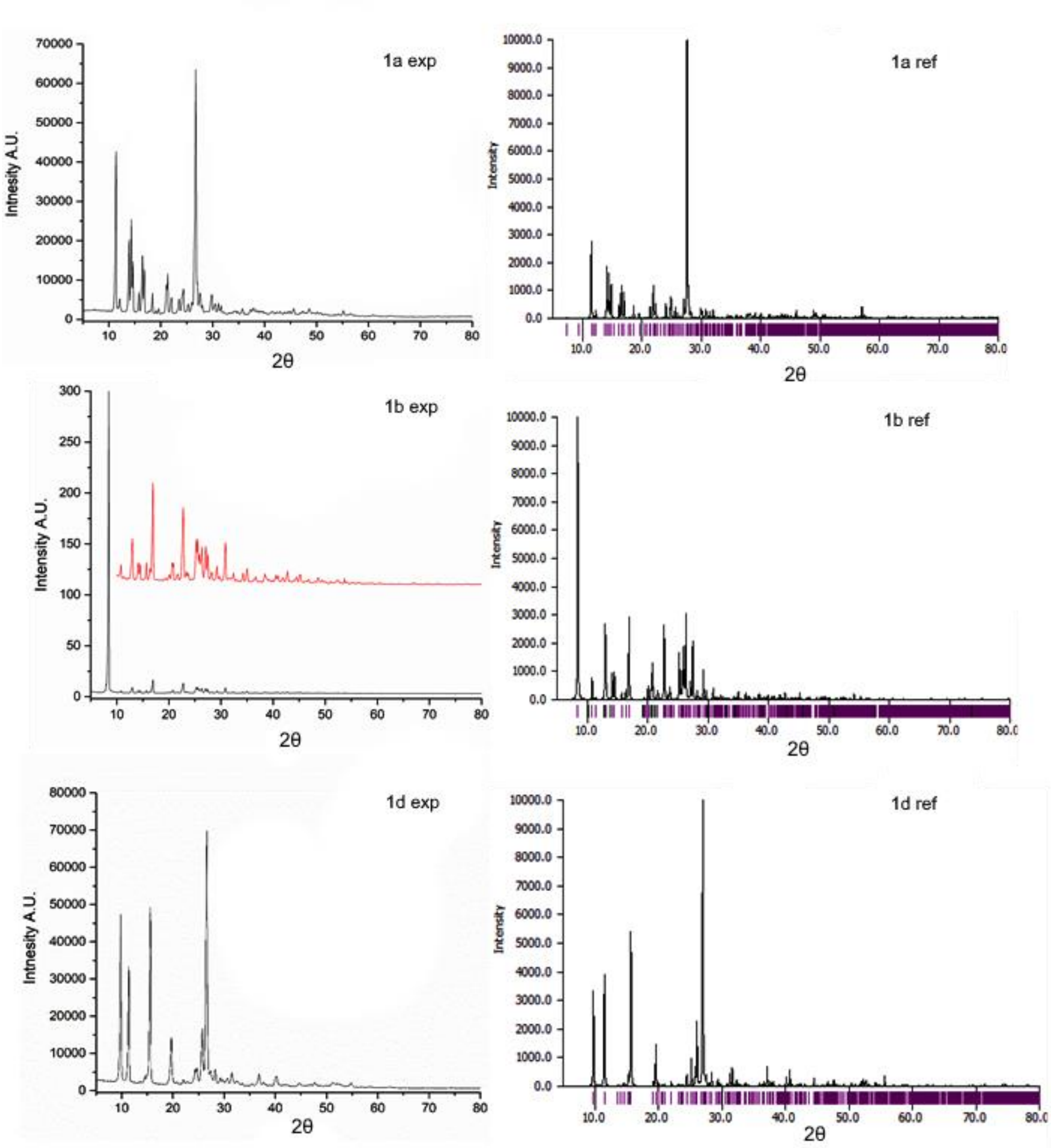

Figure S17.

Absorption and emission spectra of compounds 1a-d and 2a-d.

Quantum yield of compounds 1a-d and 2a-d

The quantum yields in solution and solid were obtained by a SC-30 module that has a xenon lamp and integrating sphere (Direct excitation method), which relates the absorbed and emitted photons. For each compound an excitation wavelength was applied to obtain the quantum yield. All measurements were carried out in triplicate. The fluorescence quantum yield (Φ), was determined by the direct excitation method and using following equation (1):

$$\eta_{DEx} = \frac{E_B}{S_A - S_B}$$

η= Absolute fluorescence quantum yield
S_A= Peak excitation reference (dissolvent)
S_B= Peak excitation sample
E_B= Peak emission sample

Theoretical calculations.

Table S1. TD-DFT parameters determined for the 3-acetyl-2H-chromen-2-one (**1a**-**d**) compounds. Data are calculated at aug-cc-pVDZ/TD-PBE0(CPCM) level of theory.

Comp.	Solvent	λ_{abs} (nm)	P.M.O.T	% Contribution to the energy	Gap P.M.O.T (eV)	Absorbance (a.u)	Oscillator strength (f)
**1a**	THF	293.7	HOMO-1 - LUMO	66.9	4.89	0.15	0.42
			HOMO - LUMO	22.9			
	Acetonitrile	294	HOMO-1 - LUMO	66.1	4.87	0.15	0.44
			HOMO - LUMO	23.8			
	Toluene	292.3	HOMO - LUMO	20.5	4.49	0.15	0.39
			HOMO-2 - LUMO	11.8			
			HOMO - LUMO+1	5.3			
**1b**	THF	409.7	HOMO-1	1.6		0.11	0.64

Comp.	Solvent	λ_{abs} (nm)	P.M.O.T	% Contribution to the energy	Gap P.M.O.T (eV)	Absorbance (a.u)	Oscillator strength (*f*)
			- LUMO+1				
			HOMO - LUMO	93.3	3.65		
	Acetonitrile	410.6	HOMO-1 - LUMO+1	1.5		0.11	0.65
			HOMO - LUMO	93.3	3.61		
	Toluene	402.4	HOMO-1 - LUMO+1	1.9		0.11	0.65
			HOMO - LUMO	93.1	3.73		
**1c**	THF	321.3	HOMO-1 - LUMO	9.0		0.14	0.54
			HOMO - LUMO	84.0	4.29		
	Acetonitrile	321.4	HOMO-1 - LUMO	9.4		0.14	0.55
			HOMO - LUMO	83.6	4.28		
	Toluene	320.7	HOMO-2 - LUMO	8.5		0.14	0.53
			HOMO - LUMO	84.7	4.31		
**1d**	THF	327.6	HOMO-1 - LUMO	5.3		0.13	0.60
			HOMO - LUMO	88.2	4.24		
	Acetonitrile	327.8	HOMO-1 - LUMO	5.3		0.13	0.60
			HOMO - LUMO	88.1	4.23		
	Toluene	326.7	HOMO-2 - LUMO	5.2		0.11	0.58
			HOMO - LUMO	88.4	4.26		

P.M.O.T.= Principal Molecular Orbital Transitions

Table S2. TD-DFT parameters determined for the 3-(phenylhydrazone)-chromen-2-one (**2a**-**d**) compounds. Data are calculated at aug-cc-pVDZ/TD-PBE0(CPCM) level of theory.

Comp.	Solvent	λ_{abs} (nm)	P.M.O.T	% Contribution to the energy	Gap P.M.O.T (eV)	Absorbance (a.u)	Oscillator strength (*f*)

**2a**	THF	538.2	HOMO – LUMO	93.2	3.00	0.08	0.60
		281.5	HOMO-3 – LUMO	32.4	5.06	0.16	0.18
			HOMO – LUMO+2	20.1			
	Acetonitrile	522.5	HOMO – LUMO	93.3	3.10	0.08	0.61
		292.3	HOMO-1 – LUMO	41.6	4.06	0.15	0.21
			HOMO – LUMO+2	32.5			
	Toluene	562.3	HOMO – LUMO	93.2	2.92	0.08	0.61
		301.2	HOMO – LUMO+2	30.1	4.83	0.15	0.19
			HOMO-1 – LUMO	27.9			
**2b**	THF	519.5	HOMO – LUMO	93.2	3.07	0.08	0.78
		349.9	HOMO-1 – LUMO	48.2	4.08	0.13	0.78
			HOMO – LUMO+1	43.0			
	Acetonitrile	502.1	HOMO – LUMO	93.1	3.10	0.09	0.82
		350.8	HOMO-1 – LUMO	55.5	4.06	0.12	0.75
			HOMO – LUMO+1	35.1			
	Toluene	550.6	HOMO – LUMO	93.4	2.99	0.08	0.73
		350.4	HOMO – LUMO+1	68.1	4.29	0.13	0.74
			HOMO-1 – LUMO	24.3		0.13	0.74
**2c**	THF	404.4	HOMO-1 – LUMO+1	90.3	3.77	0.11	0.60
		360.6	HOMO-2 – LUMO	82.0	4.26	0.12	0.50
	Acetonitrile	399.7	HOMO-1 – LUMO+1	85.6	3.79	0.11	0.57
		360.7	HOMO-2 – LUMO	80.8	4.21	0.12	0.54
	Toluene	407.3	HOMO-1 – LUMO+1	84.7	3.73	0.11	0.69
		356.9	HOMO-2 – LUMO	84.1	4.38	0.12	0.40
**2d**	THF	531.2	HOMO – LUMO	93.3	3.07	0.08	0.61
		305.5	HOMO-1	69.5	4.58	0.14	0.45

		– LUMO				
Acetonitrile	514.4	HOMO – LUMO	93.3	3.11	0.08	0.62
	305.6	HOMO-1 – LUMO	75.0	4.59	0.14	0.42
Toluene	558.6	HOMO – LUMO	93.2	2.99	0.08	0.60
	317.4	HOMO – LUMO+2	62.2	4.75	0.14	0.35

1. Edinburgh Instruments, FLS980 Series Reference Guide, Integrating sphere for measurements of fluorescence quantum yields and spectra reflectance.
